# An apparently non-swinging tentorium in the Diplopoda (Myriapoda): comparative morphology of the tentorial complex in giant pill-millipedes (Sphaerotheriida)

**DOI:** 10.3897/zookeys.741.21909

**Published:** 2018-03-07

**Authors:** Leif Moritz, Thomas Wesener, Markus Koch

**Affiliations:** 1 Zoologisches Forschungsmuseum Alexander Koenig, Leibniz Institute for Animal Biodiversity, Section Myriapoda, Adenauerallee 160, 53113 Bonn, Germany; 2 Institute of Evolutionary Biology and Ecology, University of Bonn, An der Immenburg 1, 53121 Bonn, Germany; 3 Senckenberg Gesellschaft für Naturforschung, Dept. Information Technology and Biodiversity Informatics, Senckenberganlage 25, 60325 Frankfurt am Main, Germany

**Keywords:** Arthrosphaeridae, micro-CT, 3D-reconstruction, swinging tentorium, volvation

## Abstract

The presence of a swinging tentorium is a key apomorphy of Myriapoda, but this character has been studied in detail in only few species. Here the tentorium, i.e., the peristomatic skeleton of the preoral chamber, is comparatively studied in three species of the millipede order Sphaerotheriida Brandt, 1833. Since dissections of the fragile tentorial components proved to be difficult, despite the large head size, they were analysed mainly in situ via micro-computed tomography. Our results confirm previous observations of large differences in the tentorial construction in the giant pill-millipedes compared to chilognathan diplopods. The tentorium of Sphaerotheriida consists of a curved, plate-like epipharyngeal bar with distal projections, an elongate and thin hypopharyngeal bar, and a plate-like triangular posterior process; a transverse bar is absent. Only seven muscles attach at the tentorium in giant pill-millipedes, including two antennal muscles and two muscles of the gnathochilarium. Within the order Sphaerotheriida, the composition of the tentorium and its muscular equipment seems to be conserved, except for some variability in the shape of the epipharyngeal bar. As the transverse bar has been considered essential for the mobility of the tentorium in myriapods, its absence in Sphaerotheriida may indicate that their tentorium is not capable of performing a swing. Loss of tentorial mobility may also pertain to the order Glomerida Brandt, 1833, inferred here from the absence of a posterior process. An apparently immobile tentorium in Glomerida and Sphaerotheriida can straightforwardly be correlated with transformations of the head related to their ability of volvation. The different transformations of the tentorium, here hypothesised to cause immobility, may support current assumptions that the ability of volvation evolved convergently in Glomerida and Sphaerotheriida. This conclusion, however, still requires more detailed studies of the head anatomy in Glomerida and Glomeridesmida Cook, 1895.

## Introduction

Recent molecular studies (Gai et al. 2006, Regier et al. 2010, Miyazawa et al. 2014, Fernández et al. 2016) as well as studies combining molecular and morphological data (Lee et al. 2013, Giribet et al. 2001) consistently retrieve the Myriapoda as a monophylum. Unique synapomorphies shared by its taxa Chilopoda, Symphyla, Pauropoda, and Diplopoda, however, are sparse. The most striking character in favour of myriapod monophyly is the so-called swinging tentorium, i.e. an apparently mobile skeleton of the head supporting the preoral chamber and movements of the mandibles (Edgecombe and Giribet 2002, Koch 2003, Edgecombe 2004, Shear and Edgecombe 2010, Edgecombe 2011, Koch et al. 2015). The tentorial complex consists of sclerotised exoskeletal bars and endoskeletal processes (Koch 2003, Koch 2015). This complex provides stability to the largely membranous epi- and hypopharynx and serves as muscle attachment sites. The tentorium is considered essential for the movement of the mandibles and the gnathochilarium, although the mechanism is not yet understood (Manton 1964, Fechter 1961, Koch 2015). In general the tentorium of the Diplopoda is composed of four parts: the exoskeletal (1) hypopharyngeal bar, (2) the epipharyngeal bar, (3) the transverse bar, and (4) the endoskeletal posterior process (sensu Koch 2003). Associated with the tentorial complex is an additional sclerite, the nebententorium (sensu Attems 1926, Verhoeff 1928, Koch 2015) or hypopharyngeal lateral sclerite (sensu Wilson 2002), also serving as an attachment site for musculature (Verhoeff 1928). Details on the structure of the tentorium and its musculature have been described for only few representatives of the Diplopoda. Four descriptions for members of the order Sphaerotheriida date back more than 100 years, and three of them (vom Rath 1886, Silvestri 1903, Attems 1926) describe the state for *Sphaeropoeus* Brandt, 1833 (Zephroniidae Gray, 1843). There is only one description for the Arthrosphaeridae Jeekel 1974 by Verhoeff (1928) for *Arthrosphaera dentigera* Verhoeff, 1930. Recently, the tentorium of *Zoosphaerium bemanevika* Sagorny & Wesener, 2017 from Madagascar was visualised in 3D using volume renderings of a µCT scan (Sagorny and Wesener 2017), but it was not described in detail. The Arthrosphaeridae are the second largest family with 119 species and 4 genera within the order Sphaerotheriida. The Arthrospaeridae have a very interesting biogeography with *Arthrosphaera* from the Indian sub-continent, and the three genera *Sphaeromimus*, *Zoosphaerium* and *Microsphaerotherium* Wesener & VandenSpiegel, 2007 only known from Madagascar (Wesener and VandenSpiegel 2009, Wesener et al. 2010). All descriptions indicate that the tentorium in the Sphaerotheriida deviates markedly from the pattern described for other myriapods. In order to reveal the deviating characteristics more comprehensively, the tentorium of three representatives of the Arthrosphaeridae genera *Arthrosphaera* Pocock, 1895, *Sphaeromimus* de Saussure & Zehntner, 1902 and *Zoosphaerium* Pocock, 1895 are described and compared.

## Materials and methods

Vouchers are stored in natural history collections of the Zoological Research Museum A. Koenig (**ZFMK**) and the California Academy of Sciences Herbarium (**CAS**). One head of *Zoosphaerium* sp. was used for light microscopy of the skeletal components with a Keyence VHX 700 digital stack imaging system. For this purpose the mandibles were removed with micro-scissors and the head bisectioned by slicing along the mouth with a razor blade. Micro-CT scans were taken from the heads of the three giant pill-millipede species *Arthrosphaera brandtii* (Humbert, 1865) (ZFMK MYR 06265), *Sphaeromimus kalambatritra* Moritz & Wesener, 2017 (CAS ENT 9058301) and *Zoosphaerium bemanevika* Sagorny & Wesener, 2017 (ZFMK MYR 6144), all belonging to the family Arthrosphaeridae Jeekel, 1974. The heads were dissected and critical point dried (CPD) after dehydration via an ascending ethanol series. X-ray micro-computed tomography (µCT) was performed with a SKYSCAN 1272 (Bruker microCT, Kontich, Belgium), using the following settings: source voltage = 60 kV, source current = 166 μA , exposure = 915 ms, rotation of 180° in rotational steps of 0.2°, frame averaging = 6, random movement = 15 px, filter = Al 0.25 mm. Isotropic voxel resolution varied in the following manner: *Arthrosphaera brandtii*: 5.99 µm; *Sphaeromimus kalambatritra*: 7.86 µm; *Zoosphaerium bemanevika*: 7.99 µm. Reconstruction and thermal drift correction was performed in NRecon 1.7.0.4 (Bruker microCT, Kontich, Belgium). Reduction of the data size by scaling to 50 % and conversion from 16- to 8-bit greyscale, and the adjustment of contrast and brightness was performed in IMAGE J 1.50e (Schneider et al. 2012). The resulting image stacks are deposited in MorphoBank as Project 2795 (http://morphobank.org/permalink/?P2795) Automated segmentation with subsequent manual corrections and 3D visualisation of the studied structures was performed in ITK-SNAP 3.6.0 (Yushkevich et al. 2006). Terminology follows Koch (2015) for components of the endoskeleton, and Wilson (2002) for the musculature. Illustrations and figure plates were prepared with Adobe Photoshop CS2 and Adobe Illustrator CS2.

## Results

### Skeletal elements of the tentorium in the Sphaerotheriida

**Figure 1.**
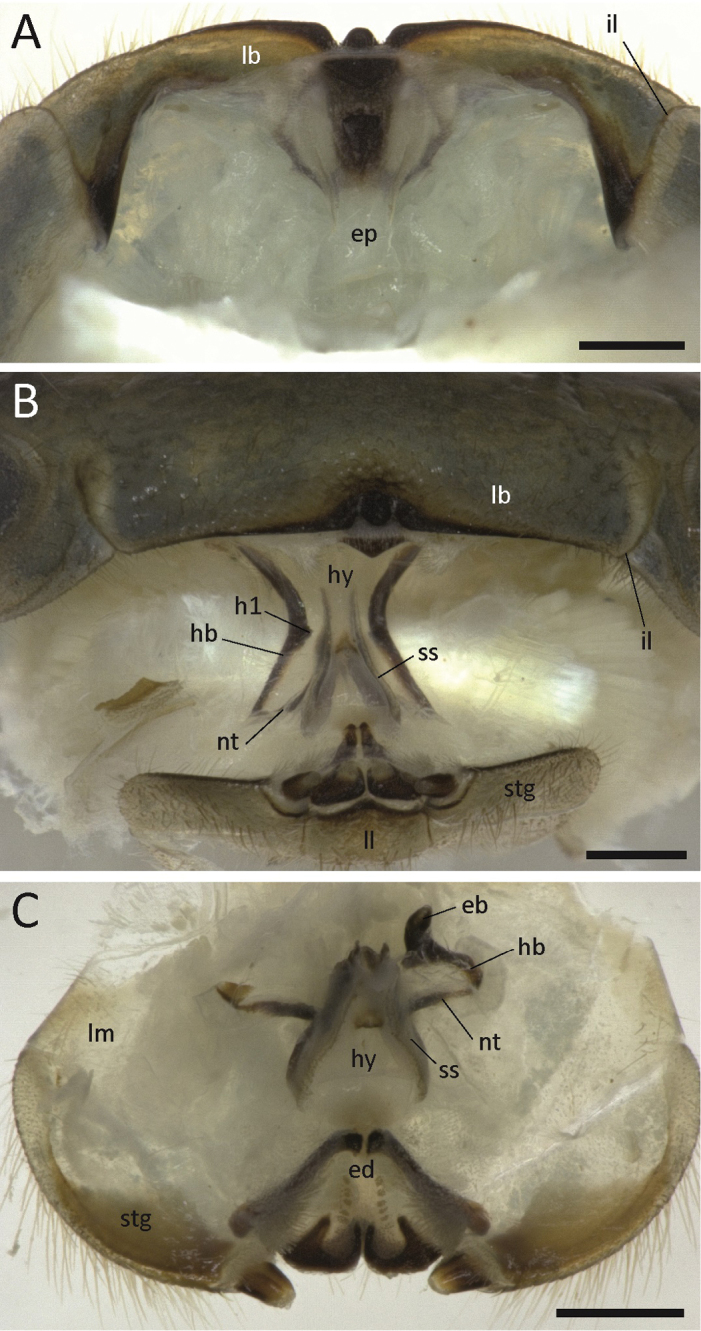
*Zoosphaerium* sp., light micrographs of peristomatic structures. **A** Epipharynx, showing absence of the tentorial transverse bar **B** Preoral chamber, frontal view on hypopharynx (mandibles removed) **C** Hypopharynx and endochilarium, dorsal view (hypo- and epipharyngeal bar of right tentorium broken off). Scale bars: 500 µm. **Abbreviations:** eb = epipharyngeal bar of left tentorium; **ed** = endochilarium; **ep** = epipharynx; **h1** = projection of hypopharyngeal bar; **hb** = hypopharyngeal bar of tentorium; **hy** = hypopharynx; **il** = incisura lateralis; **lb** = labrum; **ll** = lamella lingualis; **lm** = lamella-mentum; **nt** = nebententorium; **ss** = suspensorial sclerite; **stg** = stipes of gnathochilarium.

**Figure 2.**
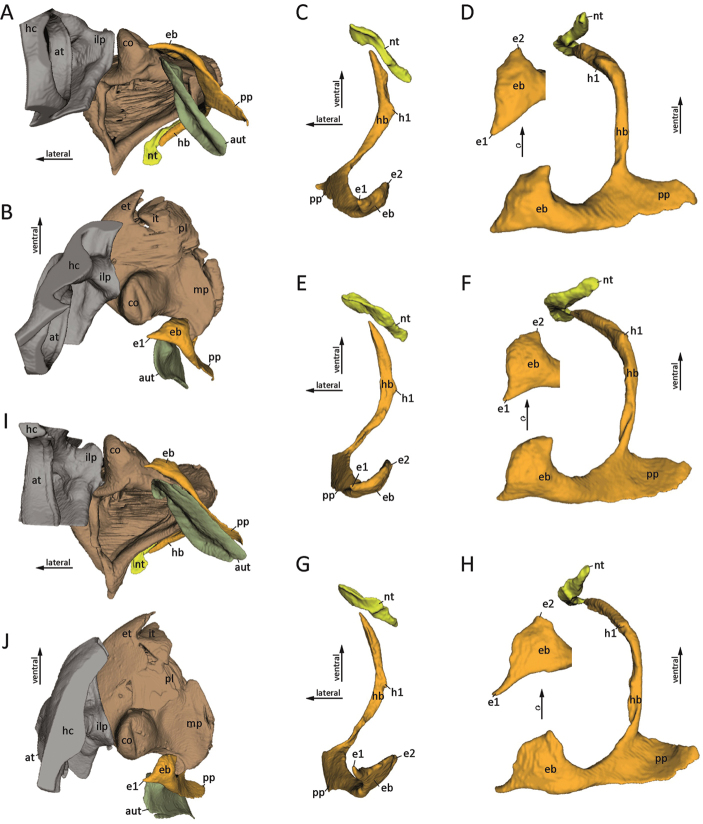
The tentorial complex of the Sphaerotheriida, 3D visualization. GREY = Head capsule; BROWN = mandible; ORANGE = tentorium; YELLOW = nebententorium; OLIVE = außententorium. **A–D** *Arthrosphaera brandtii* (Humbert, 1865), ZFMK MYR6265 **E, F, I, J** *Sphaeromimus kalambatritra*, CASENT 9058301 **G, H** *Zoosphaerium bemanevika* Sagorny & Wesener, 2017, ZFMK MYR6144. **A** tentorial complex and its association with the mandibular gnathal lobe and the head capsule of *A. brandtii*, dorsal view **B** same as A frontal view **C** tentorial complex of *A. brandtii*, frontal view **D** same as C medial view, with rotated epipharyngeal bar **E** tentorial complex of *S.* sp., frontal view **F** same as E medial view, with rotated epipharyngeal bar **G** tentorial complex of *Z. bemanevika*, frontal view **H** same as G medial view, with rotated epipharyngeal bar **I** tentorial complex and its association with the mandibular gnathal lobe and the headcapsule of *S.* sp., dorsal view **J** same as I, frontal view. **Abbreviations**: **at** = antennal socket; **aut** = mandibular gnathal lobe sclerite (außententorium); **co** = condylus of mandible; **e1** = lateral projection of epipharyngeal bar; **e2** = medial projection of epipharyngeal bar; **eb** = epipharyngeal bar; **et** = external tooth; **h1** = projection of hypopharyngeal bar; **hb** = hypophayrangeal bar; **hc** = head capsule; **ilp** = projection arising from incisura lateralis; **it** = internal tooth; **mp** = molar plate; **nt** = nebententorium; **pl** = pectinate lamellae; **pp** = posterior process.

A connection of the tentorium to the head capsule by a transverse bar (sensu Koch 2003) is missing in the three analysed species, despite the presence of an incisura lateralis (Fig. 1A). The paired tentorial complex consists of only four major parts: the epipharyngeal bar (Fig. 1C, eb), the hypopharyngeal bar (Fig. 1B, hb), the posterior process (Fig. 2, pp), which forms a single tripartite sclerite (the tentorium sensu stricto) along the mouth opening, and the separate nebententorium (nt; hypopharyngeal lateral sclerite sensu Wilson 2002) located on the hypopharynx (Fig. 1B, hy).

**(1) The epipharyngeal bar**:

The plate-like epipharyngeal bar (eb) is in connection with the wall of the epipharynx (Fig. 2A–J, ep). The distal part of the epipharyngeal bar is a triangular plate with one slightly curved lateral projection (e1) and a stout median projection (Fig. 1C, D, e2). The shape of the projections of the epipharyngeal bar is variable within the Arthrosphaeridae (Fig. 2D, F, H): the lateral projection (e1) is rather stout and short in *Sphaeromimus kalambatritra* (Fig. 2F), more elongate in *Arthrosphaera brandtii* (Fig. 2D) and long, slender and curved in *Zoosphaerium bemanevika* (Fig. 2H). The median projection (e2) is triangular in *A. brandtii* (Fig. 2D) and *S. kalambatritra* (Fig. 2F), and rectangular in *Z. bemanevika* (Fig. 2H). The distal part of the epipharyngeal bar (eb) is curved, following in shape the curvature of the mandible condyles (Fig. 2A, B, I, J, co) in all analysed specimens. The proximal part of the epipharyngeal bar (eb) is a rectangular plate, which is wider in *A. brandtii* (Fig. 2D) and *S. kalambatritra* (Fig. 2F), while it is more slender in *Z. bemanevika* (Fig. 2H).

**(2) The hypopharyngeal bar**:

In the three analysed species, the epipharyngeal bar (eb) of the tentorium (Fig. 2A–J) passes over into the hypopharyngeal bar (hb) posteriorly to the pharyngeal opening. The hypopharyngeal bar is elongate and rod-like (Fig. 2A–J, hb). The bar is strongly curved inward and extends ventrally on the hypopharynx towards the gnathochilarium where it is associated to the nebententorium (nt) via a membranous connection (Fig. 2C, E, G). A small cone-shaped medial projection (h1) close to the center of the hypopharyngeal bar is present (Fig. 2C–H), pointing to the hypopharyngeal suspensorial sclerites (Fig. 1B, ss) (Stützgerüst sensu Attems 1926; Verhoeff 1928). Although the hypopharyngeal bar of *A. brandtii* (Fig. 2C) is slightly shorter in relation to its width than in *S. kalambatritra* (Fig. 2E) and *Z. bemanevika* (Fig. 2G), its general rod-like appearance can be seen in all analysed species.

**(3) The posterior process**:

The posterior process (pp) is a large triangular plate projecting posteriorly into the head capsule parallel to the mandibular gnathal lobe sclerite (sensu Wilson 2002; äußeres Tentorium sensu Voges 1916, Attems 1926; Außententorium sensu Seifert 1932; (German for "outer tentorium")) (Fig. 2A, B, I, J, aut). It arises from the transition point between the epi- (eb) and hypopharyngeal bars (hb). There is no variation in the shape of the posterior process (pp) within the studied Arthrosphaeridae (Fig. 2C–H).

**(4) The nebententorium**:

The nebententorium (nt) is a short, flat sclerite parallel to the distal portion of the hypopharyngeal bar (hb) of the tentorium (Fig. 2A, B, I, J, YELLOW). It bypasses the distal tip of the hypopharyngeal bar slightly and broadens, forming an articulation with the tentorium (Fig. 2C–H).

### The connection of the mandible to the tentorium in Arthrosphaeridae

**Figure 3.**
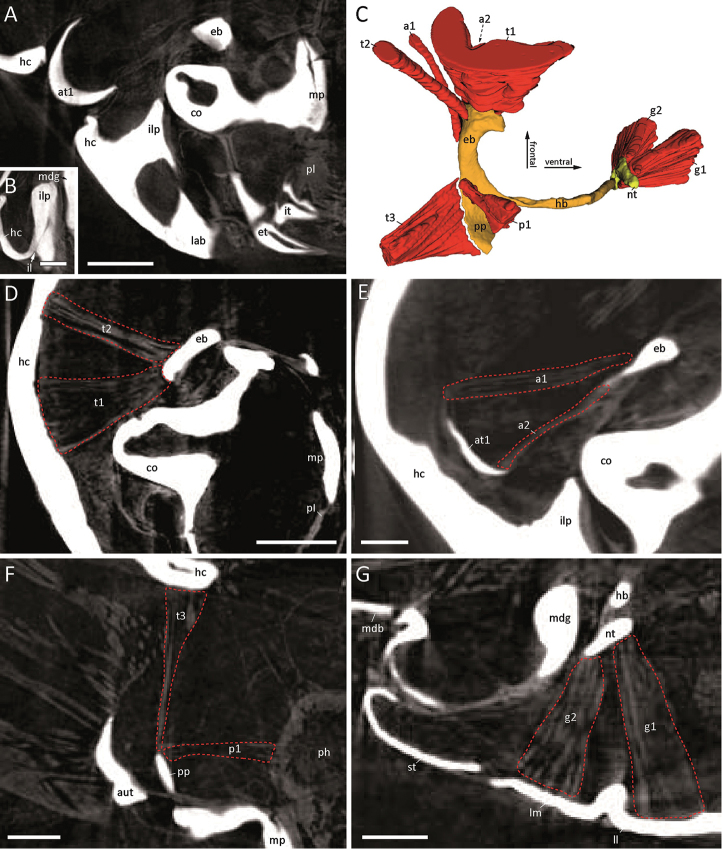
Head musculature of *Sphaeromimus kalambatritra*. **A, B, D–G** micro-CT images **C** 3D segmentation. **A** Arrangement of mandible, tentorium and head capsule, frontal section **B** Incisura lateralis in detail, frontal section **C** 3D segmentation of the isolated tentorium and its muscles, medial view **D, E** Muscles of the epipharyngeal bar, fronto-medial view **F** Muscles of the posterior process, frontal section **G** Muscles of the nebententorium, frontal section. Top is frontal, left is lateral. Scale bars: **A, D** 1000 µm **B, E–G** 500 µm **C** not to scale. **Abbreviations**: **a1** = lateral antennal muscle (m.); **a2** = anterior antennal m.; **at1** = first antennomere; **co** = condyle of mandibular gnathal lobe; **eb** = epipharyngeal bar; **et** = external tooth of mandible, **g1** = lamella lingualis m.; **g2** = lamello-mentum m.; **aut** = mandibular gnathal lobe sclerite (außententorium); **hb** = hypophayrangeal bar; **hc** = head capsule; **il** = incisura lateralis; **ilp** = projection arising from incisura lateralis; **it** = internal tooth of mandible; **lab** = labrum; **ll** = lamella lingualis of gnathochilarium; **lm** = lamello-mentum; **mdb** = mandibular base; **mdg** = mandibular gnathal lobe; **mp** = molar plate; **nt** = nebententorium; **p1** = pharyngeal dilator m.; **ph** = pharynx; **pl** = pectinate lamellae of mandible; **pp** = posterior process; **st** = stipes of gnathochilarium; **t1** = anterior tentorial m.; **t2** = dorsal tentorial m.; **t3** = posterior tentorial m.

The strong condylus (co) of the mandibular gnathal lobe (Fig. 2A, B, I, J, BROWN) is not in direct contact with the tentorium, but medially faces the epipharyngeal bar (Fig. 2A, B, I, J, eb). Lateral of the condylus arises a sclerotised socket-shaped projection (Fig. 3A, ilp) from the incisura lateralis (il) of the head capsule (Fig. 3B, hc). The mandibular condylus hence appears to be encompassed by both the epipharyngeal bar and the sclerotised projection of the incisura lateralis.

### Musculature of the tentorium in Arthrosphaeridae

The tentorial complex of the Arthrosphaeridae is associated with a set of seven muscles (Fig. 3C), which do not vary in the studied species. The proximal part of the epipharyngeal bar (eb) gives rise to the anterior tentorial muscle (t1), which inserts on the anterior part of the head, and to the dorsal tentorial muscle (t2), which inserts medial of the antennal socket (Fig. 3D). The posterior tentorial muscle (t3) inserts on the whole length of the posterior margin of the posterior process (pp) and originates from the postoccipital flange close to the transition to the collum (Fig. 3C, F). The lateral antennal muscle (a1) originates from the posterior margin of the transitional area between the epipharyngeal bar (eb) and the posterior process (pp), anteriorly of t3, and inserts on the posterior margin of the first antennomere (at1). The anterior antennal muscle (a2) inserts on the anterior margin of the first antennomere (at1) and originates from the epipharyngeal bar (eb) lateral of t3 (Fig. 3E). Median to t3, the pharyngeal dilator muscle (p1), which inserts on the lateral pharyngeal wall (ph), originates from the frontal anterior portion of the posterior process (Fig. 3F, pp). The nebententorium (nt) gives rise to a muscle (g1) inserting medially on the lamella lingualis (ll) of the gnathochilarium. Another muscle (g2) of the gnathochilarium passes from the lamello-mentum (lm) to the posterior surface of the nebententorium (nt) lateral to g1 (Fig. 3G).

## Discussion

### Structure of the tentorium in the Sphaerotheriida

The tentorium of the three studied representatives of Sphaerotheriida shows the same basic structure (Fig. 2C–H), as already described by vom Rath (1886), Silvestri (1903) and Attems (1926) for *Sphaeropoeus*, and by Verhoeff (1928) for *Arthrosphaera dentigera*. Vom Rath (1886) stated that the tentorium of the Sphaerotheriidae *Sphaerotherium* Brandt, 1833 resembles that of the Zephroniidae *Sphaeropoeus* Brandt, 1833, without a detailed description. Therefore, the general structure of the tentorium seems to be highly conserved within the Sphaerotheriida.

The most striking character of the giant pill-millipede tentorium is the absence of the transverse bar (Fig. 1C), which is present in all other millipede orders as far as known. Although the transverse bar differs among millipedes in its shape and articulation to the head capsule, it is known to be present in the Polyxenida (Koch 2003), Glomerida (vom Rath 1886, Silvestri 1903, Voges 1916), Julida (vom Rath 1886, Silvestri 1903, Voges 1916, Attems 1926, Verhoeff 1928, Fechter 1961), Spirobolida (vom Rath 1886, Snodgrass 1950), Spirostreptida (vom Rath 1886, Silvestri 1903, Manton 1964), Polydesmida (vom Rath 1886, Silvestri 1903, Verhoeff 1928, Seifert 1932, Snodgrass 1950), Chordeumatida (vom Rath 1886, Verhoeff 1928), Callipodida (Verhoeff 1928) and Platydesmida (Koch 2015). The state of the transverse bar (or the tentorium in general) for Glomeridesmida, Stemmiulida, Siphoniulida and most Colobognatha has not been documented yet. A transverse bar can nevertheless be assumed for the ground pattern of Diplopoda. Its reduction can be viewed as a derived state of the Sphaerotheriida.

### Impact on tentorial mobility

The general function of the transverse bar is the connection of the tentorial complex to the head capsule at the incisura lateralis (= clypeal notch), around which the tentorium is deemed to perform its swinging movements (Manton 1964). Furthermore, the transverse bar is the insertion site for tentorial protractor muscles (Manton 1964, Wilson 2002). Along with the reduction of the transverse bar in Sphaerotheriida, the mobility of the tentorium must have undergone tremendous changes and must differ from the mechanism assumed by Fechter (1961) and Manton (1964) for Juliformia, as the muscular equipment of the tentorium in Sphaerotheriida (Fig. 3 C) varies greatly from that of Juliformia. While Wilson (2002) reported 13 muscles attached to the tentorium in Spirostreptida, Sphaerotheriida only maintain seven muscles (Fig. 3C). In Sphaerotheriida, the anterior tentorial muscle and the dorsal tentorial muscles originating on the epipharyngeal bar (Fig. 3D), as well as the posterior tentorial muscle and pharyngeal dilator muscles originating from the posterior process (Fig. 3F) correspond to the state in Juliformia (Wilson 2002). The tentorial protractor muscles, however, apparently shifted their position from the transverse bar (as described by Wilson 2002) to the distal part of the epipharyngeal bar. Further differences concern the antennal muscles that arise from the posterior process of the tentorium in Juliformia, but from the epipharyngeal bar in Sphaerotheriida (Fig. 3E). In Sphaerotheriida, contrarily to Juliformia (Silvestri 1903, Manton 1964, Wilson 2002), no mandibular muscles arise from the tentorium. In the Sphaerotheriida all muscles of the mandibular base instead originate from the transverse mandibular tendon and cranial wall, respectively. Among the three gnathochilarial muscles arising from the nebententorium in Juliformia, only two are present in Sphaerotheriida, i.e., the one (g1) inserting on the lamella-mentum, and the one (g2) inserting on the lamellae linguales (Fig. 3G). The muscle extending from the gnathochilarial stipes to the nebententorium is apparently reduced in giant pill-millipedes. This reduction of gnathochilarial muscles is likely due to strong modifications of the gnathochilarium in Sphaerotheriida, which are considered as autapomorphies of this taxon (Wesener 2016).

Despite these differences, the main muscles considered essential for movements of the tentorial complex are present. Manton (1964) states that the movement of the tentorium in Spirostreptida results from the tension of the protractor tentorii (anterior tentorial muscle sensu Wilson 2002, t1) and the depressor tentorii, which correspond to the lamella lingualis muscle (g1) and the lamello-mentum muscle (g2) of Sphaerotheriida.

The differences in the composition of the tentorium and in its muscular equipment might not only be correlated with the absence of the tentorial transverse bar, but also with the presence of a strong condylus on the mandibular gnathal lobes of Sphaerotheriida that unquestionably impacts on the mandibular mechanism. In Juliformia, the connection of the transverse bar to the incisura lateralis is deemed to fix a swing of the tentorium, causing the mandibular gnathal lobe to abduct (Fechter 1961, Manton 1964). In Sphaerotheriida, we propose that abduction of the mandibular gnathal lobes instead is caused by forces exerted from the epipharyngeal bar on the condylus of the gnathal lobes. The shape of the epipharyngeal bar indicates that it interlinks to the notch present on the condylus (Fig. 2A, J) to cause abduction of the gnathal lobe by pushing its condylus into the projection of the incisura lateralis. The theory about this movement is further corroborated by the shape correlation between the shape of the notch and the curvature of the epipharyngeal bar. The curvature of the epipharyngeal bar is more shallow in *A. brandtii*, where the notch forms a larger plateau (Fig. 2A) than in *S. kalambatritra*, in which the notch of the condylus is more strongly curved (Fig. 2I). The projections of the incisura lateralis and the epipharyngeal bar appear to form an anchor around which the mandibular gnathal lobe rotates during its abduction. According to this interpretation, the tentorial protractor and retractor muscles likely do no longer cause the tentorium to swing. The modified muscles instead likely serve to stabilise the tentorium in a position enabling the condylus of the gnathal lobe to rotate between the incisura lateralis and the epipharyngeal bar.

### Correlations of the shape of the tentorium with volvation: a comparison with Glomerida

The tentorium of Sphaerotheriida contributes more characters to the list of head modifications that likely correlate with adaptations to volvation (see, e.g., Golovatch 2003, Blanke and Wesener 2014, Tuf et al. 2016). These adaptations include a reduction of the head lumen and coincident size reduction or entire loss of endoskeletal formations (surveyed by Koch 2015). Among Pentazonia, both Sphaerotheriida and Glomerida are able to roll themselves up into a ball, but their tentorium displays different transformations: as inferred from *Glomeris marginata*, the transverse bar is primarily maintained (e.g., Voges 1916, Dohle 1964), albeit with a remarkably loose connection to the head capsule. However, unlike in Sphaerotheriida, in the Glomerida the posterior process of the tentorium seems to be absent (Koch 2015). Since the posterior process provides the origin of the tentorial retractor muscle (t3), the loss of the posterior process may indicate that the tentorium in Glomerida is, as in Sphaerotheriida, no longer capable of performing swinging movements. The different modifications of the tentorium, the reduction of the transverse bar in Sphaerotheriida and of the posterior process in Glomerida, corroborate the view that anatomical adaptations to volvation are non-homologous (Sierwald and Bond 2007, their Supplemental Appendix 1), i.e., that volvation evolved convergently in Glomerida and Sphaerotheriida. This view is particularly supported by recent molecular analyses (Regier et al. 2005, Fernández et al. 2016), as well as by characters of the gnathochilarium (Wesener and Van den Spiegel 2009) favouring a sister group relationship between Glomerida and Glomeridesmida over the traditional classification of Glomerida and Sphaerotheriida in the taxon Oniscomorpha. Our ongoing studies focus on a detailed comparison of the cephalic musculature in Glomerida and Glomeridesmida to test the hypothesis of convergent loss of a swinging tentorium in correlation with convergent gain of volvation in Glomerida and Sphaerotheriida.

### The tentorium as a taxonomic character inside Sphaerotheriida

Although the general appearance of the tentorium is conserved within Arthrosphaeridae there are some differences in details. These mainly concern the epipharyngeal bar, with its projections varying in their shape and length (Fig. 2D, F, H). Slight variations in shape are also displayed by the hypopharyngeal bar and the nebententorium (Fig. 2C, E, G). The tentorium of *Sphaeromimus* (Fig. 2E, F) is more similar to the state in *Arthrosphaera* (Fig. 2C, D) than in *Zoosphaerium* (Fig. 2G, H). This corresponds to the interrelationship within Arthrosphaeridae retrieved by Wesener et al. (2010) from molecular analyses, according to which the Malagasy genus *Sphaeromimus* is more closely related to the Indian genus *Arthrosphaera* than to the other Malagasy genera *Zoosphaerium* and *Microsphaerotherium*. The structure and shape of the tentorium accordingly might also serve as an informative character not only for phylogenetic reconstructions, but also for taxonomic studies, which can be assessed quite rapidly with high-throughput techniques like µCT and automated 3D-segmentation. However, not investigated yet were the changes in the structure of the tentorium in different life stages of millipedes and intraspecific variations. We recommend that internal characters should more often be considered in taxonomic descriptions.

## Conclusions

The reduction of the transverse bar of the tentorial complex as well as the presence of the mandible condyles in Sphaerotheriida must have an enormous impact on the mandibular abduction, resulting in a probably non-swinging tentorium. The reduction of the transverse bar in Sphaerotheriida is probably correlated to the volvation and suggests a convergent evolution of volvation in the pentazonian orders Sphaerotheriida and Glomerida. In Glomerida the posterior process of the tentorial complex is reduced as an adaptation to volvation. This could furthermore support a previously suggested (Regier et al. 2005, Fernández et al. 2016, Wesener and van den Spiegel 2009) closer relationship between Glomerida and Glomeridesmida. Furthermore the tentorium offers taxonomic characters to distinguish at least genera. Therefore we recommend considering internal characters more often in taxonomic descriptions. Despite its importance as apomorphy, supporting the monophyly of Myriapoda, and its functional role, the tentorial complex is largely understudied and the knowledge on it throughout the Myriapoda is only fragmentary. This study can be seen as first step towards a broader assessment of the tentorial complex in the Diplopoda.

## References

[B1] Attems C (1926) Myriapoda. In: Kükenthal W Krumbach T (Eds) Handbuch der Zoologie. Eine Naturgeschichte der Stämme des Tierreiches. Progoneata, Chilopoda, Insecta. Walter de Gruyter & Co, Berlin and Leipzig, 1–402.

[B2] Blanke A Wesener T (2014) Revival of forgotten Characters and modern imaging Techniques help to produce a robust phylogeny of the Diplopoda (Arthropoda, Myriapoda). Arthropod structure & development 43(1): 63–75. https://doi.org/10.1016/j.asd.2013.10.003 24184600 10.1016/j.asd.2013.10.003

[B3] Brandt IF (1833) Tentaminum quorundam monographicorum Insecta Myriapoda Chilognatha Latreillii spectantium Prodromus. Bulletin de la Société impériale des naturalistes de Moscou 6: 194–209.

[B4] Cook OF (1895) Introductory note on the families of Diplopoda. The Craspedosomatidae of North America. Annals of the New York Academy of Sciences 9: 1–100. https://doi.org/10.1111/j.1749-6632.1896.tb55430.x

[B5] De Sassure HLF Zehntner L (1902) Myriapodes de Madagascar. In: Grandidier A (Ed.) Histoire physique, naturelle et politique de Madagascar. Mémoires du Muséum national d’Histoire naturelle 27(53), 1–356.

[B6] Dohle W (1964) Die Embryonalentwicklung von *Glomeris marginata* (Villers) im Vergleich zur Entwicklung anderer Diplopoden. Zoologische Jahrbücher. Abteilung für Anatomie und Ontogenie der Tiere Abteilung für Anatomie und Ontogenie der Tiere 81: 241–310.

[B7] Edgecombe GD (2004) Morphological data, extant Myriapoda, and the myriapod stem-group. Contributions to Zoology 73(3): 207–252.

[B8] Edgecombe GD (2011) Phylogenetic relationships of Myriapoda. In: Minelli A (Ed.) The Myriapoda, Volume 1. Brill, Leiden, 1–20. https://doi.org/10.1163/9789004188266_002

[B9] Edgecombe GD Giribet G (2002) Myriapod phylogeny and the relationships of Chilopoda. In: Llorente Bousquets JE Morrone JJ (Eds) Biodiversidad, taxonomía y biogeografía de artrópodos de México: Hacia una síntesis de su conocimiento. Prensas de Ciencias, Universidad Nacional Autónoma de México, Mexico-City, Mexico, 143–168.

[B10] Fechter H (1961) Anatomie und Funktion der Kopfmuskulatur von *Cylindroiulus teutonicus*. Zoologische Jahrbücher, Abteilung für Anatomie und Ontogenie der Tiere: 479–582.

[B11] Fernández R Edgecombe GD Giribet G (2016) Exploring Phylogenetic Relationships within Myriapoda and the Effects of Matrix Composition and Occupancy on Phylogenomic Reconstruction. Systematic Biology 65(5): 871–889. https://doi.org/10.1093/sysbio/syw041 27162151 10.1093/sysbio/syw041 PMC4997009

[B12] Gai YH Song DX Sun HY Zhou KY (2006) Myriapod Monophyly and Relationships among Myriapod Classes based on nearly complete 28S and 18S rDNA Sequences. Zoological Science 23(2): 1101–1108. https://doi.org/10.2108/zsj.23.1101 17261924 10.2108/zsj.23.1101

[B13] Giribet G Edgecombe GD Wheeler WC (2001) Arthropod phylogeny based on eight molecular loci and morphology. Nature 413: 157–161. https://doi.org/10.1038/35093097 11557979 10.1038/35093097

[B14] Golovatch SI (2003) A review of the volvatory Polydesmida, with special Reference to the patterns of Volvation (Diplopoda). African Invertebrates 44(1): 39–60.

[B15] Gray EJ (1842) Myriapoda In: Jones TR, Todd RB (Eds) Cyclopedia of anatomy and physiology 3: 544–560.

[B16] Humbert A (1865) Essai sur les Myriapodes de Ceylan. Mémoires de la Société de Physiques et d’Histoire naturelle de Genève 18: 1–63.

[B17] Jeekel CAW (1974) The group taxonomy and geography of the Sphaerotheriida (Diplopoda). Symposia of the Zoological Society of London 32: 41–52.

[B18] Koch M (2003) Monophyly of the Myriapoda? Reliability of current arguments. African Invertebrates 44(1): 137–153.

[B19] Koch M (2015) Diplopoda – general morphology. In: Minelli A (Ed.) The Myriapoda, Volume 2. Brill, Leiden, 7–68. https://doi.org/10.1163/9789004188273_003

[B20] Koch M Schulz J Edgecombe GD (2015) Tentorial mobility in centipedes (Chilopoda) revisited: 3D reconstruction of the mandibulo-tentorial musculature of Geophilomorpha. Zookeys 510: 243–267. https://doi.org/10.3897/zookeys.510.8840 10.3897/zookeys.510.8840 PMC4523777 26257547

[B21] Lee MSY Soubrier J Edgecombe GD (2013) Rates of phenotypic and genomic evolution during the Cambrian explosion. Current Biology 23(19): 1889–1895. https://doi.org/10.1016/j.cub.2013.07.055 24035543 10.1016/j.cub.2013.07.055

[B22] Manton SM (1964) Mandibular mechanisms and the evolution of arthropods. Philosophical Transactions of the Royal Society of London B 247: 1–183. https://doi.org/10.1098/rstb.1964.0001

[B23] Miyazawa H Ueda C Yahata K Su ZH (2014) Molecular phylogeny of Myriapoda provides insights into evolutionary patterns of the mode in post-embryonic development. Scientific reports 4: 1–9. https://doi.org/10.1038/srep04127 10.1038/srep04127 PMC3927213 24535281

[B24] Moritz L Wesener T (2017) Integrative description of two new species of Malagasy chirping giant pill-millipedes, genus Sphaeromimus (Diplopoda: Sphaerotheriida: Arthrosphaeridae). European Journal of Taxonomy 381: 1–25. https://doi.org/10.5852/ejt.2017.381

[B25] Pocock RI (1895) XLIII.-Report upon the Chilopoda and Diplopoda obtained by PW Bassett-Smith, Esq., Surgeon RN, and JJ Wallcer, Esq., RN, during the cruise in the Chinese Seas of HMS ‘Penguin,’Commander WU Moore commanding. Journal of Natural History 15(88): 346–369. https://doi.org/10.1080/00222939508677895

[B26] Regier JC Wilson HM Shultz JW (2005) Phylogenetic analysis of Myriapoda using three nuclear protein-coding genes. Molecular Phylogenetics and Evolution 34(1): 147–158. https://doi.org/10.1016/j.ympev.2004.09.005 15579388 10.1016/j.ympev.2004.09.005

[B27] Regier JC Shultz JW Zwick A Hussey A Ball B Wetzer R Martin JW Cunningham CW (2010) Arthropod relationships revealed by phylogenomic analysis of nuclear protein-coding sequences. Nature 463: 1079–1083. https://doi.org/10.1038/nature08742 20147900 10.1038/nature08742

[B28] Sagorny C Wesener T (2017) Two new giant pill-millipede species of the genus *Zoosphaerium* endemic to the Bemanevika area in northern Madagascar (Diplopoda, Sphaerotheriida, Arthrosphaeridae). Zootaxa 4263(2): 273–294. https://doi.org/10.11646/zootaxa.4263.2.4. 28609869 10.11646/zootaxa.4263.2.4

[B29] Schneider CA Rasband WS Eliceiri KW (2012) NIH Image to ImageJ: 25 years of image analysis. Nature Methods 9: 671–675. https://doi.org/10.1038/nmeth.2089 22930834 10.1038/nmeth.2089 PMC5554542

[B30] Seifert B (1932) Anatomie und Biologie des Diplopoden *Strongylosoma pallipes*. Zoomorphology 25(2): 362–507. https://doi.org/10.1007/BF00446714

[B31] Shear WA Edgecombe GD (2010) The geological record and phylogeny of the Myriapoda. Arthropod Structure & Development 39: 174–190. https://doi.org/10.1016/j.asd.2009.11.002 19944188 10.1016/j.asd.2009.11.002

[B32] Sierwald P Bond JE (2007) Current status of the myriapod class Diplopoda (millipedes): taxonomic diversity and phylogeny. Annual Review of Entomology 52: 401–420. https://doi.org/10.1146/annurev.ento.52.111805.090210 10.1146/annurev.ento.52.111805.090210 17163800

[B33] Silvestri F (1903) Classis Diplopoda Anatome: Pars I, Segmenta, Tegumentum, Musculi. In: Berlsese A (Ed.) Acari, Myriapoda et Scorpiones huscque in Italia reperta, Portici, 1–272.

[B34] Snodgrass RE (1950) Comparative Studies on the Jaws of Mandibulate Arthropods. Smithsonian Miscellaneous Collections 116(1): 1–85.

[B35] Tuf IH Cmielova L Sipos J (2016) Conglobation as a defensive behavior of pill millipedes (Diplopoda: Glomerida). Acta Societatis Zoologicae Bohemicae 80: 39–44.

[B36] Verhoeff KW (1928–1932) Diplopoda I. Bronns Klassen und Ordnungen des Tierreichs, vol. 5. Akademischer Verlag, Leipzig, 1–1071.

[B37] Voges E (1916) Myriapodenstudien. Zeitschrift für Wissenschaftliche Zoologie 116: 75–135.

[B38] vom Rath O (1886) Beitraege zur Kenntnis der Chilognathen. Doctoral thesis, Strasbourg, France, Kaiser-Wilhelms-Universität Strasburg.

[B39] Wesener T (2016) The Giant Pill-Millipedes, order Sphaerotheriida – An annotated species catalogue with morphological atlas and list of apomorphies (Arthropoda: Diplopoda). Bonn Zoological Bulletin (Supplementum 63): 1–104.

[B40] Wesener T van den Spiegel D (2007) *Microsphaerotherium ivohibiensis*, a new genus and species of Giant-Pill Millipedes from Madagascar (Diplopoda, Sphaerotheriida, Arthrosphaerinae). Journal of Afrotropical Zoology 3: 153–160.

[B41] Wesener T van den Spiegel D (2009) A first phylogenetic analysis of Giant Pill-Millipedes (Diplopoda: Sphaerotheriida), a new model Gondwanan taxon, with special emphasis on island gigantism. Cladistics 25: 545–573. https://doi.org/10.1111/j.1096-0031.2009.00267.x 10.1111/j.1096-0031.2009.00267.x 34879594

[B42] Wesener T Raupach MJ Sierwald P (2010) The origins of the giant pill-millipedes from Madagascar (Diplopoda: Sphaerotheriida: Arthrosphaeridae). Molecular Phylogenetics and Evolution 57(3): 1184–1193. https://doi.org/10.1016/j.ympev.2010.08.023 20813191 10.1016/j.ympev.2010.08.023

[B43] Wilson HM (2002) Muscular anatomy of the millipede *Phyllogonostreptus nigrolabiatus* (Diplopoda: Spirostreptida) and its bearing on the millipede “thorax”. Journal of Morphology 251(3): 256–275. https://doi.org/10.1002/jmor.1087 11835363 10.1002/jmor.1087

[B44] Yushkevich PA Piven J Hazlett HC Smith RG Ho S Gee JC Gerig G (2006) User-guided 3D active contour segmentation of anatomical structures: Significantly improved efficiency and reliability. Neuroimage 31(3): 1116–28. https://doi.org/10.1016/j.neuroimage.2006.01.015 16545965 10.1016/j.neuroimage.2006.01.015

